# Evaluating the Contribution of Complex International Research-for-Development Programmes to the Sustainable Development Goals

**DOI:** 10.1057/s41287-022-00573-7

**Published:** 2023-01-06

**Authors:** Arlene Lu-Gonzales, Takuji W. Tsusaka, Sylvia Szabo, Reuben M. J. Kadigi, Camilla Blasi Foglietti, Seree Park, Zoe Matthews

**Affiliations:** 1 School of Environment, Resources and Development, Asian Institute of Technology, Pathum Thani, 12120 Thailand; 2 Department of Environmental Science, College of Agriculture, Food and Sustainable Development, Mariano Marcos State University, 2906 Batac City, Ilocos Norte Philippines; 3 Department of Social Welfare Counseling, College of Future Convergence, Dongguk University, Seoul, 04620 South Korea; 4 College of Economics and Business Studies, Sokoine University of Agriculture (SUA), P.O. BOX 3007, Morogoro, Tanzania; 5 UN Environmental Programme World Conservation Monitoring Centre (UNEP_WCMC), 219 Huntingdon Road, Cambridge, CB3 0DL UK; 6 Division of Social Statistics and Demography, University of Southampton, Southampton, SO17 1BJ UK

**Keywords:** SDG evaluation, AHP, Sustainable development, Impact mapping, Interaction analysis

## Abstract

While evaluation of research-to-policy projects is a fundamental aspect of measuring the impact of new knowledge, limited studies have examined evaluation methods in such projects, as well as how the evaluation can generate learning to facilitate the progress towards the Sustainable Development Goals (SDGs). This study conducted a systematic literature review and found that the most commonly used methods for SDG contribution evaluation were Analytical Hierarchy Process (40.4%), Fuzzy TOPSIS (13.2%) and ELECTRE and SPADE Methodology (3.5% each). Ranking analysis was undertaken to determine priorities among the six “Big Wins” as defined for the UKRI-GCRF Trade Hub Project, as a case, where the ranking was exercised by the project partners across the globe. Results revealed that “nature and social factors” was better considered in international trade agreements as the priority (36.4%) among others. Moreover, among the four “mechanisms” of the project, “knowledge, networks, and connectivity” was ranked as the top priority (56.9%), followed by “capacity building” (28.5%), “metrics, tools and models” (7.2%), and “improving the knowledge base” (4.6%). Mapping and evaluation revealed that the Big Wins of the Trade Hub contributed to ten out of the 17 SDGs. The most fulfilled goals were SDG 12 (Sustainable Consumption and Production), SDG 15 (Life on Land), and SDG 2 (Zero Hunger) in descending order. Furthermore, interaction analysis of the core SDGs revealed both synergy and tradeoff between different outputs. The research articles reviewed for this paper showed no gold standard framework for assessing international development projects against the SDGs. Further research should develop a tool to capture holistic and synergistic contributions of the target outcomes of projects to sustainable development.

## Résumé

Bien que l'évaluation des projets sur le lien entre recherche et politique soit un aspect fondamental de la mesure de l'impact des nouvelles connaissances, peu d’études ont examiné les méthodes d'évaluation de tels projets, ainsi que la manière dont l'évaluation peut générer un apprentissage pour faciliter la progression vers les objectifs de développement durable (ODD). Cette étude a mené une revue systématique de la littérature et a constaté que les méthodes les plus couramment utilisées pour l'évaluation de la contribution aux ODD étaient le processus d’analyse hiérarchique (40,4%), la méthode TOPSIS floue (13,2%), et les méthodes ELECTRE et SPADE (3,5% chacune). Une analyse par classement a été entreprise pour déterminer les priorités parmi les six « Grands Succès» tels que définis pour le projet UKRI-GCRF Trade Hub, par exemple, où le classement a été réalisé par les partenaires du projet à travers le monde. Les résultats ont révélé que les « facteurs naturels et sociaux» étaient mieux considérés dans les accords commerciaux internationaux comme la priorité (36,4%), parmi d’autres. De plus, parmi les quatre « mécanismes» du projet, « les connaissances, les réseaux et la connectivité» ont été classés comme la première priorité (56,9%), suivis du « renforcement des capacités» (28,5%), « les mesures, les outils et les modèles» (7,2%) et « améliorer la base de connaissances» (4,6%). La cartographie et l'évaluation ont révélé que les Grands Succès du projet Trade Hub ont contribué à dix des 17 ODD. La contribution aux objectifs était la plus importante, par ordre décroissant, pour l'ODD 12 (Consommation et production durables), l'ODD 15 (Vie terrestre) et l'ODD 2 (Faim zéro). En outre, l'analyse des interactions des principaux ODD a révélé à la fois une synergie et un compromis entre les différents produits. Les études examinées pour cet article n'ont montré aucun cadre de référence pour évaluer les projets de développement international par rapport aux ODD. Des études supplémentaires devraient être conduites pour développer un outil permettant de mesurer les contributions holistiques et synergiques des résultats cibles des projets au développement durable.

## Introduction

Programme evaluation plays a crucial role in determining the outcomes and impacts of complex international research-for-development (R4D) projects. Evaluation can clarify whether the research is effective and what specific change it contributes to by building evidence of the benefits of policies, programmes, and strategies. Previous studies [e.g. Austrian Development Agency (ADA) 2007] emphasise the importance of evaluation because of its critical role in ensuring transparency, iterative learning, and fostering communication. In this light, evaluation of R4D programmes becomes a way of strengthening the capacity for implementation of research that can achieve real-life development outcomes. Sebesvari et al. (2016) indicate that such international development research is focused on addressing complex phenomena and multidimensional issues (e.g. tackling poverty, combating climate change, and reducing inequalities). Their focus on grand challenges is inherently linked to the United Nation (UN)’s Sustainable Development Goals (SDG); yet R4D projects are not necessarily aligned with specific SDG targets, indicators, and sub-indicators, and thus, their programme evaluation tends to miss the potential opportunity for monitoring progress towards the UN Agenda 2030. This paper explored the potential of connections across these two levels evaluation in practice.

The Agenda 2030 is a complex and ambitious framework for addressing global challenges (Miola and Schiltz 2019; D’Errico et al. 2020; Gennari and D’Orazio 2020), which includes 17 SDGs and 169 targets for economic, social, and environmental prosperity for all people. The existing SDG evaluation framework identified a need for practitioners to apply multiple indicators with a basic assumption that “one-size-fits-all” measurement would likely yield conflicting results in the process of aggregating the indicators, leading to a plethora of data that are potentially not usable or useful (Davis et al. 2015). Current evaluation approaches fall short of the need to build an evidence base for action (Allen et al. 2018). The SDG framework is so broad and complex that regional and national priorities and circumstances need to be taken into consideration when setting up useful evaluation frameworks. In so doing, it is difficult to identify targets and indicators where there is inadequate progress, which necessitates adjustments to the existing indicator-based assessment. Prior studies imply that assessment that suits a particular local context poses challenges to SDG evaluation due to the gaps and mismatches between theoretical indicators and local conditions (Yonehara et al. 2017).

Several countries have performed their comprehensive SDG evaluation using different approaches. Finland, for example, focused on their sustainable development policies and cross-administrative foreign policies aimed at understanding the preconditions and mechanisms of implementation (Anukka et al. 2019). Nigeria focused on three specific goals (SDGs 1, 3, and 4) to assess whether their strategic plans achieved the targets (D’Errico et al. 2020). A similar approach was adopted by Costa Rica where the evaluation was focused on the achievement of their three priority points of entry, namely, fighting poverty, substantiable production and consumption, and sustainable infrastructure and communities (Griggs et al. 2020). Their evaluation approach was a mapping of the SDG targets and indicators against the national indicators to determine the progress of the country towards achieving the SDGs. Evaluation can differ in scope and purposes, and thus, assessment of integration is one of the critical components in conducting SDG evaluation. Moreover, there is no single way to implement SDG evaluation due to the disparity between countries, affecting the setting of indicators.

Recently, development activities are encouraged to devote attention to the sustainability aspect along with usefulness and effectiveness of their projects (Maier et al. 2016). However, the key challenge in evaluation of contributions to the SDG lies in finding an approach or method that meets the evaluation requirement. Unlike previous methods, evaluation for the SDG implies the integration of the three interdependent dimensions of sustainable development, namely: social, economic, and environment dimensions. Despite the existence of various approaches and methods according to evaluation types, a more comprehensive, harmonised, and integrated method would enable cross-evaluation of different SDG interventions (Adou 2017).

As such, this paper underscores the need for more research, better measurements, and more informed evaluation strategies for R4D projects, based on monitoring progress towards all SDG targets, to help formulate, implement, and adjust development plans. Furthermore, these strategies should ensure that all stakeholders are accountable for achieving the SDGs (Schneider et al. 2019; Gennari and D’Orazio 2020). Moreover, policies and plans, as well as MEL (monitoring, evaluation, and learning) efforts failed to recognise the systematic nature of the SDGs, resulting in silos that ignore interactions between and around projects or programmes (Ofir et al. 2019). In this regard, this paper views creation of a strong interface between research and policy as imperative. As research can play crucial roles in addressing the SDGs, key stakeholders, including policymakers, would benefit enormously from better understanding of research. However, while methods for capturing complex interconnections between SDGs are developed, potential contribution of research remains complex and elusive.

The objective of this paper is to provide a comprehensive review and mapping of SDG evaluation tools and methods applicable for international development research projects. It aims to examine the applicability of the most frequently used evaluation methods to international R4D projects’ contribution to SDG progress. The UKRI-GCRF Trade Hub project is used as a case in assessing this applicability.

## Conceptual Framework

A great deal of research is carried out each year on subjects that are directly relevant to policy and practice in international development. Substantial resources are being devoted to development research, and many organisations are involved in one way or another. Translating goals into an actionable roadmap requires a robust monitoring and evaluation process to determine the relevance and level of achievements, effectiveness, efficiency, impacts, and sustainability assessment, accounting for social, economic, and environmental implications, positive and negative alike.

Literature indicates that complex international development research is bound to address complex phenomena and multidimensional issues that provide useful lessons to monitoring for the Agenda 2030 as well as the formulation and implementation of post-2020 biodiversity framework. Mainstream literature of the Agenda 2030, for example, acknowledges that the Agenda 2030 is complex and an ambitious development framework setting and the basic assumption of one-size-fits-all measurements has attracted critiques from scholars in sustainable development (Georgeson and Maslin 2018, p. 12; Miola and Schiltz 2019, p. 5; D’Errico et al. 2020; Gennari and D’Orazio 2020).

To evaluate the progress of development projects towards the SDGs, it is crucial to conduct sustainability assessment using standardised approaches and criteria. With many development activities aiming to align their contributions to the SDGs, literature to date shows that limited methods have been developed and applied for sustainability assessment.

The UKRI-GCRF Trade, Development and the Environment Hub project (hereinafter referred to as the “Trade Hub”) was used as a case for in-depth analysis of the SDG evaluation. The Trade Hub is a 5-year multi-country research consortium generating evidence and suggesting practical solutions that promote sustainable trade, production, and harvesting of agricultural and wildlife resources to support livelihoods. Through collaboration with private and public sector institutions in the eight project countries, initiatives are focused on promoting efficient resource use and building resilience in the production systems. The Trade Hub promotes sustainable utilisation of ecosystem resources ensuring that partners across the different countries work with institutions and farmers in striking a balance between conserving swathes of forests and expanding farming areas. Within the value chains promoted by the Trade Hub, private sector companies are encouraged to adopt sustainable practices and achieve a fair-trade footprint, allowing upstream value chain actors such as farmers and small companies to increase their value markup. As an R4D project, the Trade Hub is configured to strengthen scientific and technical capacity of researchers and development practitioners in less developed and emerging economies to move towards sustainable patterns of consumption and production.

**Fig. 1:**
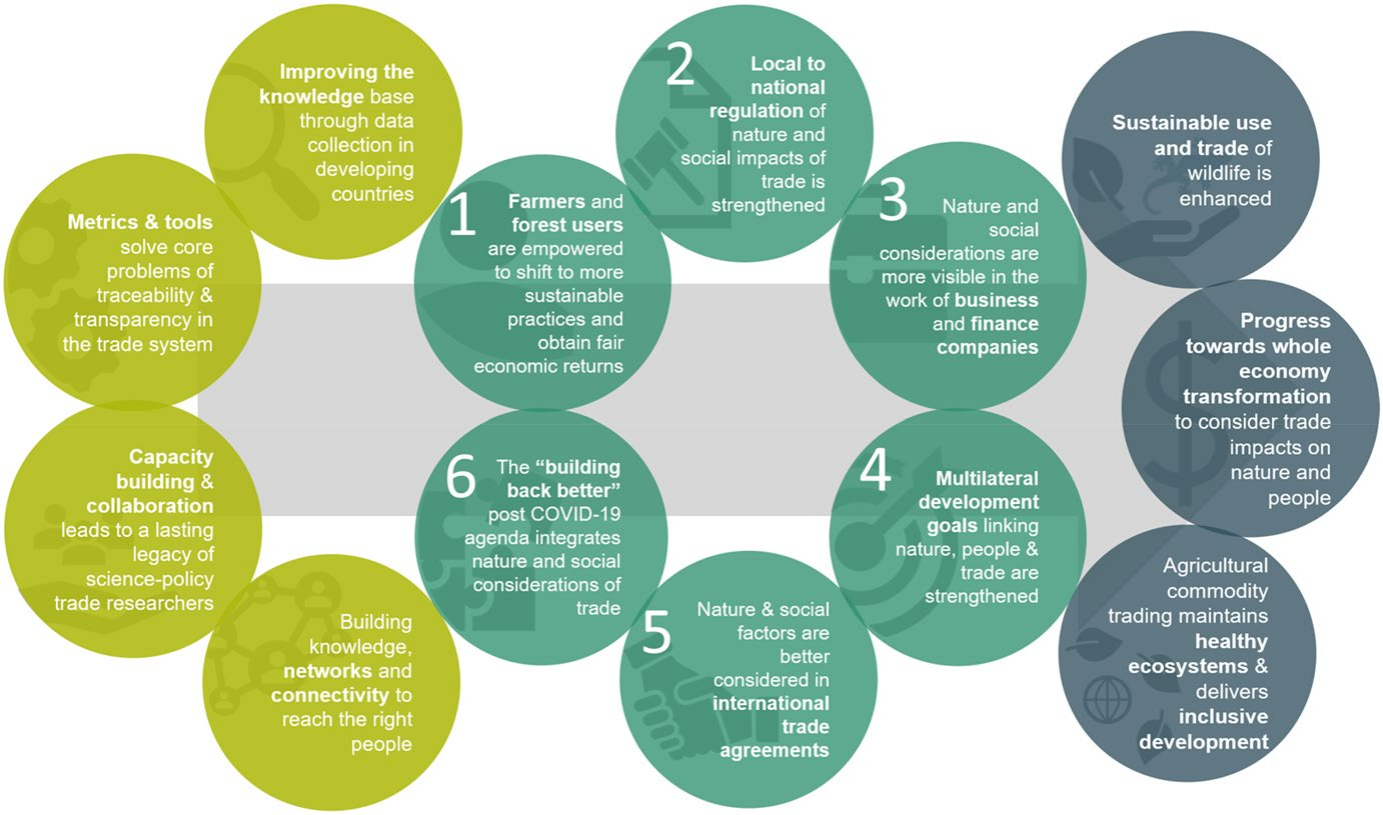
The six “Big Wins” of the Trade Hub

Figure 1 shows the schematic diagram illustrating six Big Wins (BWs), four mechanisms, and three transformational changes as defined for the Trade Hub. The project works towards the six leverage points called BWs towards which all research and development activities are ultimately oriented. These BWs encompass issues related to supply chains and trade from farmers and forest users through local to national supply chains into the regional and global trade systems as well as rules and drivers of the system. The Trade Hub seeks to provide tools and means to manage trade in sustainable manners, linking it to the international mandate as well as the UK government priorities. The four mechanisms on the left-hand side direct the goals of the Trade Hub while the three outcomes on the right contribute to the SDG targets.

**Fig. 2:**
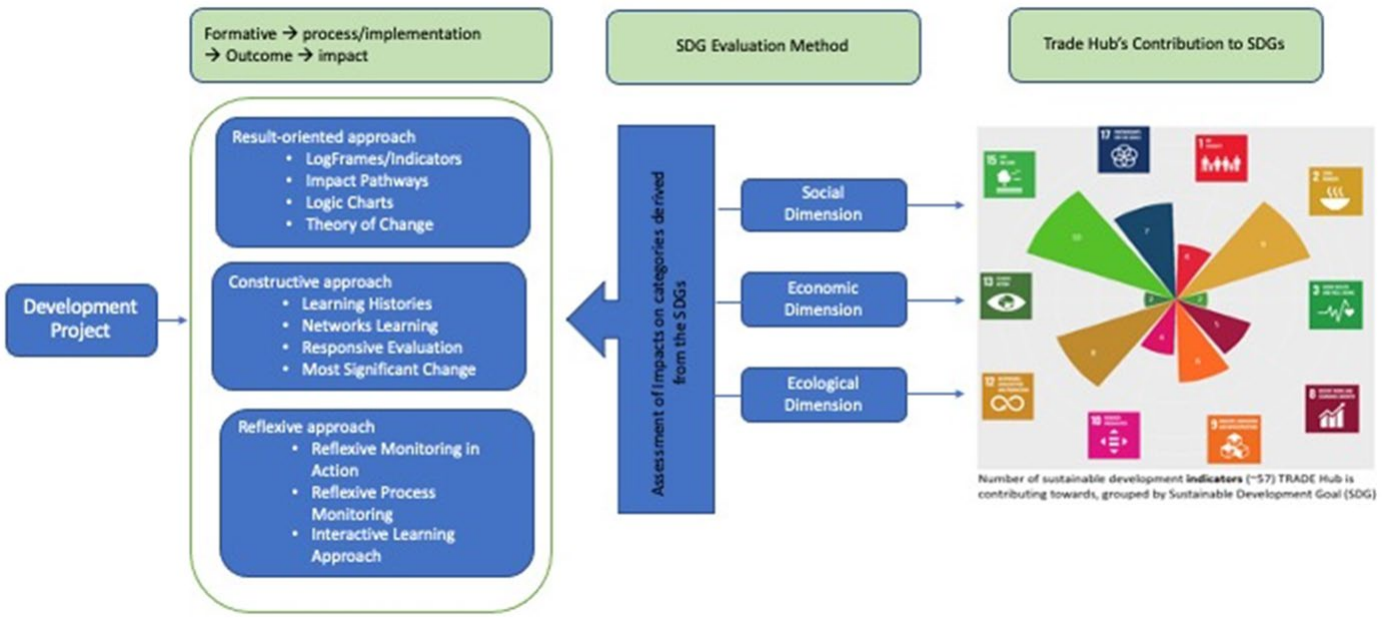
Conceptual framework of the study

Figure 2 illustrates the framework that the different approaches for impact evaluation in development projects can be simultaneously used to measure the project contribution to the SDGs, where the three dimensions of sustainability are captured. Mapping of the contributions of the BWs to the SDG was employed, and the outcomes of the partners were classified according to their alignment to the BWs. Afterwards, they were matched with corresponding SDG targets with their connection and relationship mapped.

## Methods

### Systematic Literature Review

This study applied the systematic review of literature, which is a research method designed to identify, evaluate, and interpret all available research to the research questions or topic areas, or phenomenon of interest (Seetha et al. 2021; Sousa et al. 2021). Studies included in this review were selected from the major databases, namely, Thomson Reuters Web of Science, SCOPUS, and Google Scholar. Given the subject of this paper, green literature (i.e. reports from international organisations and research institutes) was also included.

### Search Terms

To search the databases for appropriate papers, several search terms were used as shown in Table 1. Keywords were divided into three aspects: evaluation, international development research, and SDGs. The search terms were specified using the Boolean operators. OR was used to bridge any of the search terms that were similar in meaning and used interchangeably. Search terms included those related to evaluation methods (“evaluation* OR *evaluation method* OR coast* assessment*”), as well as “SDG”, “project” terms to identify research related to evaluation assessment in research-to-policy projects (“research project* OR *research program* OR *policy*”). No threshold was set for any limitations in terms of the year of publication.

**Table 1:** Search terms used in the systematic literature review

Research aspect	Alternative terms and synonyms
Evaluation	(Evaluation OR evaluation method OR evaluation methodologies OR evaluation framework OR assessment framework)
International development research	(International development research OR international development study OR international development research projects OR development projects OR development study)
SDGs	(SDG monitoring OR SDG targets OR SDG goals OR SDG aims SDG progress)

### Eligibility Criteria

Literature identified in the search was screened based on the evaluation methods adopted in the literature and the impacts of evaluated projects on the SDGs. The screening considered each study’s title, abstract, and keywords to determine whether it should be included.

Papers that belonged to one of the following attributes were screened out:

- Papers that were not relevant to international development.
- Papers that did not specify the review’s primary goals.
- Papers that were not from reputable sources.
- Papers that did not report any impact.
- Same papers in different sources
- Papers published in non-English languages.

### Quality Assessment

**Fig. 3:**
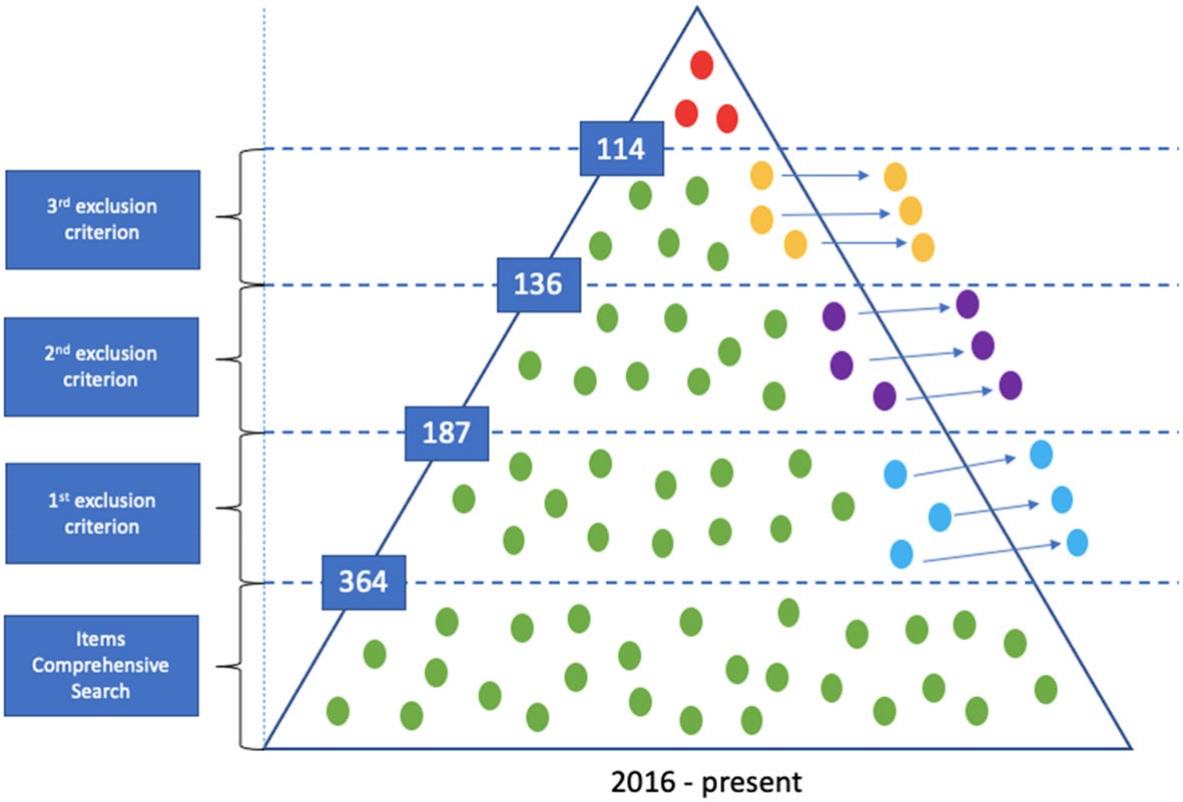
Summary of search results. *Source* Adapted from Smith et al. (2020)

The quality of the chosen papers was assessed using some criteria: whether the papers had all the required information such as funding agency, amount of funding, geographical location, evaluation methods, contribution to the SDGs, and impacts thereof; whether the topic was aligned with our intended field of study; how many times the papers had been cited by others. The papers were regarded as more reliable when the numbers of citations were larger. A flow diagram for the literature search is shown in Fig. 3. Using the general search terms, 364 published articles were found. After classifying the articles related to international development projects, 187 papers were left and further screened using the criterion evaluation methods for SDG contribution. After further classification, 136 publications were reevaluated based on the previous search criteria, and the final number of valid papers was 114.

### Research Mapping

Research mapping was used to interpose results of the systematic review findings and the Trade Hub’s contributions to the progress towards the SDGs, based on the targets, indicators, and sub-indicators. While definitions may vary, research mapping generally refers to a “review that seeks to identify, not results, but linkages” (Cooper 2016).

### In-Depth Interviews/Survey

To develop a summary of the potential SDG contribution of the Trade Hub project, the output of every partner was matched with the SDG targets. It was then circulated to all global partners using the project’s monitoring and evaluation (M&E) dashboard to receive consensus. After the summary was approved, the partners were asked to weigh (pairwise comparisons) the BWs based on their importance in the achievement of the outputs and the SDGs. Thirteen partners from five countries were participated in the survey.

### SDG Impact Mapping

Impact mapping was conducted to connect the achievement of the outputs of Trade Hub partners to the different SDG indicators in order to establish their contributions. The analysis was further extended with interaction analysis among and between the project outputs to determine the synergy and tradeoff in between. The guideline proposed by Griggs et al. (2016) was the basis in establishing the interaction (Table 2).

**Table 2:** Synergy and tradeoff in SDG evaluation

Type of interaction	Description
Cancelling (− 3)	Progress on one target automatically leads to a negative on another
Counteracting (− 2)	Progress on one target brings difficulty in progress on another
Constraining (− 1)	Progress on one target constrains the options for how to deliver on another
Neutral (0)	There is no significant link between two targets’ progress
Enabling (+ 1)	Progress on one target makes creates conditions that enable progress on another
Reinforcing (+ 2)	Progress on one target makes it easier to make progress on another
Indivisible (+ 3)	Progress on one target automatically delivers progress on another

*Source* Adapted from Griggs et al. (2016)

## Findings and Discussions

### Evaluation of Contributions to the SDGs

SDG evaluation implies the integration of the three interdependent dimensions of sustainable development as well as a number of principles and criteria necessary to achieve these goals. These make it difficult to establish a single criteria and approach in sustainability evaluation. One of the difficulties in performing SDG evaluation is the limited methods for this purpose (Pope et al. 2004).

This paper reviewed the applicability of different methods used in gauging the impacts of development projects in the context of SDG impact evaluation. To date, there is a significant focus on how to measure the impact and contributions of countries, organisations, private sectors, and public sectors towards the fulfilment of sustainable development.

The most common method used in SDG impact evaluation was the Analytical Hierarchy Process (40.4%), followed by the Fuzzy TOPSIS (13.2%). The ELECTRE and SPADE Method accounted for 3.5% each. All the other identified methods were relatively minor in SDG evaluation (Table 3).

**Table 3:** Different methods adopted in literature for SDG evaluation

SDG evaluation method	Number of articles	
	*n*	%
AHP	46	40.4
Fuzzy AHP	2	1.8
ELECTRE method	4	3.5
TOPSIS method	1	0.9
Fuzzy TOPSIS	15	13.2
SWOT analysis	2	1.8
STEEP analysis	1	0.9
SPADE methodology	4	3.5
VIKOR	1	0.9
DEMATEL	1	0.9
ANP	2	1.8
Data envelopment analysis	2	1.8
Integrated assessment models	2	1.8
Total	114	100

*AHP* Analytical Hierarchy Process; *TOPSIS* Technique for Order Performance by Similarity to Ideal Solution; *DEMATEL* Decision Making Trial and Evaluation Laboratory; *PROMETHEE* Preference Ranking Organization Method for Enrichment of Evaluations; *VIKOR* VIekriterijumsko Kompromisno Rangiranje; *ANP* Analytic Network Process; *ELECTRE* Elimination of et Choix Traduisant la Realité

### Popularity of the AHP

The multi-criteria decision method (MCDM) is relevant since problems are multidimensional, involving people, institutions, natural resources, and the environment. The MCDM is a branch of operational research aimed at finding optimal results in complex scenarios, involving various indicators, conflicting objectives, and criteria (Sousa et al. 2021). An array of MCDM methods are available, and popularly used ones are the AHP, ANP, TOPSIS, data envelopment analysis (DEA), and fuzzy decision making. The AHP, TOPSIS, DEMATEL, and VIKOR were commonly adopted to assess SDG progress in fields of energy, urban development, education, infrastructure, sanitation, and healthcare systems.

The popularity of the AHP among other MCDM for SDG evaluation is due to its ease, scalability, and the ability to capture subjective and objective evaluation measures even in complex scenarios (Londoño-Pineda et al. 2021, Ghorbanzadeh et al. 2019; Nam et al. 2019; Zhang et al. 2019). Another reason for the popularity and advantage of the AHP is its simplicity for both experts and non-experts. Its approach of a hierarchal system of criteria follows a structure while the weighing by pairwise comparisons is so reasonable that decision makers do not necessarily need to go into the mathematical details of the calculation (Szabo et al. 2021). The use of a nine-point scale for comparing criteria relative to their importance makes it easier to understand when applied to complex issues. Moreover, the AHP is flexible and allows adaptation to conditions according to the most pressing issues and needs (Londoño-Pineda et al. 2021). In addition, the analysis of sustainable development incorporates the holistic integration of the economic, social, and environmental dimensions. The use of the AHP for the indicator weighing in inter-thematic frameworks helps analyse across these three dimensions (Gan et al. 2017).

The AHP can decompose a complex problem into several simple problems, which are linked in hierarchy where criteria are ranked via pairwise comparison. The pairwise comparison allows the decision maker to deal with the prioritisation between only two options at a time, irrespective of their other options (Gompf et al. 2021). The Fuzzy TOPSIS is a simple computational procedure, easy to represent human preferences, and allows explicit tradeoffs between multiple criteria. The solution is based on the shortest distance from its ideal solution while the AHP is based on the weight for each criterion judged in pairwise comparisons and priorities are calculated.

The SDG evaluation methods have been evolving and increasingly applied since the launch of the 2030 Agenda in 2016 as a guiding principle. According to the analysis by Gompf et al. (2021) using 143 literature items, the growth rate of relevant publications was 56% in 2016–2017 and 122% in 2019–2020. The methods covered in her study were closely similar to the methods listed in Table 2.

Castor et al. (2020) identified sustainability assessment methods with a focus on frameworks and analytical tools to assess sustainability of projects from the economic, social, or environmental perspective, or a combination of these pillars. Among the many variations of tools for sustainability assessment, commonly used ones incorporating the SDGs are the Environmental Impact Assessment (EIA) (EPA 2019), Life Cycle Assessment (LCA) (Nes et al. 2007), Sustainability Impact Assessment (SIA) (OECD 2010), SDG Impact Assessment Tool (SDSN 2019), and mapping the interactions between the SDGs (Nilsson et al. 2016). These tools allow quick assessment of multiple impacts by guiding mapping exercises and fulfilling project requirements. However, these tools are either incomplete, not incorporating all aspects of sustainable development (EIA, LCA, Social-LCA, SIA), or information useful in considering a specific project (SDG Impact Assessment Tool, mapping the interactions between the SDG) is not provided (Castor et al. 2020).

Recent studies on evaluation of contributions to the SDGs of higher education (Kioupi and Voulvoulis 2020), the energy sector (Castor et al. 2020), and green building (Wen et al. 2020) developed novel frameworks to build a logical theoretical model, alignment of intended (learning) outcomes, and a realistic mapping tool as a medium for evaluating contributions. These frameworks were developed with the aim of providing a universal tool that can be used as a guide when assessing SDG contributions in the field of education and energy.

### SDG Evaluation for the Trade Hub Project

Mapping of the contributions of the BWs to the SDGs was also adopted and the outputs of every work package (WP) in the Trade Hub were classified first according to their alignment to the six BWs. Afterwards, they were matched with corresponding SDG targets with their connection mapped, which automatically organises data into relationship. The same approach was used in Costa Rica for their country evaluation of progress towards sustainable development where their data sources were linked to the SDG indicators proposed by the UN (Griggs et al. 2020).

In addition, the AHP was also employed to determine which among the BWs had the greatest contributions. A pairwise comparison among the different BWs and the ranking were used according to experts’ perception of the weight of every BW. The AHP was used to set the level of priority of the outputs contributing to the SDG indicators.

**Fig. 4:**
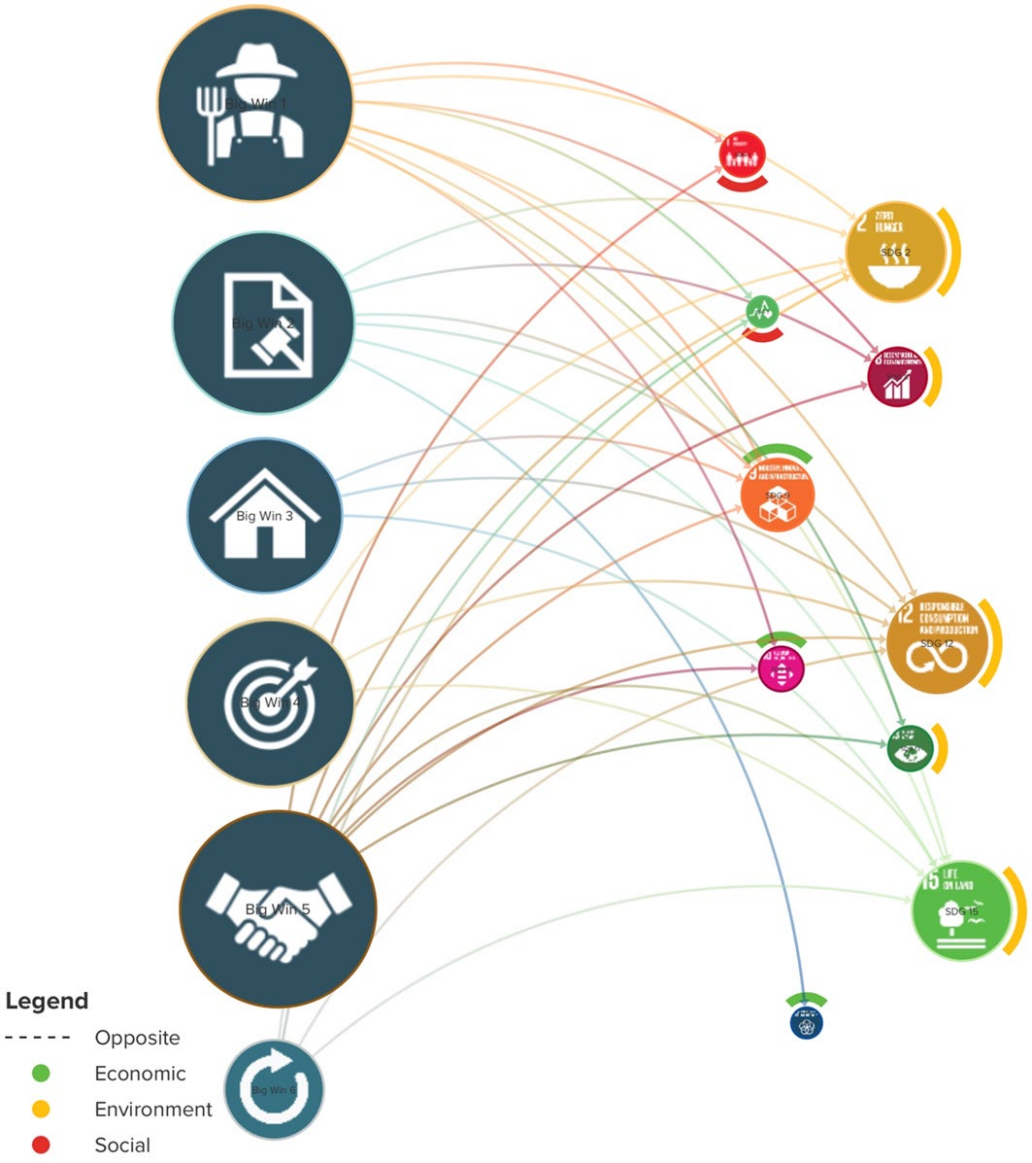
The TRADE Hub’s six Big Wins contributing to the specific SDGs

Moreover, the partners working for the Trade Hub project identified and described their activities and outputs related to sustainable development, which were individually examined and connected to the different SDG indicators. It was found that the six BWs contributed to ten of the 17 SDGs, encompassing the social goals: SDG 1 (No Poverty), SDG 2 (Zero Hunger), SDG 3 (Good Health and Well-being), SDG 10 (Reduced Inequalities), SDG 17 (Partnerships for the Goals); economic goals: SDG 1 (No Poverty), SDG 2 (Zero Hunger), SDG 8 (Decent Work and Economic Growth), SDG 9 (Industry, Innovation, and Infrastructure), SDG 17 (Partnerships for the Goals); and environmental goals: SDG 12 (Responsible Consumption and Production), SDG 13 (Climate Action), and SDG 15 (Life on Land). The result of the impact mapping is shown in Fig. 4.

#### SDG 1 No Poverty

One of the Trade Hub’s objectives is to help connect smallholder farmers and small-scale local producers to buyers in the private sector to ensure their integration in mainstream markets and benefits from trade. In this way, the local producers who are mostly low-income households are supported with innovations and technologies, which enhance their production systems, and hence, overall production of food and other tradeable agricultural commodities and wildlife species. With the effort to bring scenarios in the global, regional, and country-level markets, a better understanding of how commodities circulate in markets brings emphasis on smallholder farmers and, thus, opportunities for them to enhance their practices towards sustainable production and improving yields as well as resilience of their resources against climate-related events and other disasters. The project caters to “no poverty” by increasing opportunities for raising production and income for smallholder farmers and local producers, hence, reducing the number of people in extreme poverty.

#### SDG 2 Zero Hunger

Working with smallholder farmers and other value chain actors, the Trade Hub contributes to enhancing food production at the household level. This improves the availability and affordability of food that maximizes household consumption and utility. With this, the beneficiaries of the initiatives across the eight countries are guaranteed with the efforts towards a zero-hunger world are successful. The key value chains supported by the initiatives can unlock smallholder farmers’ potential to increase their productivity and enhance their food and nutrition security. The successful generation of new knowledge on functionality or markets is the epitome of collaborative partnership between the private and public sectors and research institutions towards developing solutions to the problems bedevilling the less developed countries.

#### SDG 3 Ensure Healthy Lives and Promote Well-Being for All at All Ages

In global trade, the Trade Hub highlights value addition to natural resource products grown and produced by smallholder farmers. As their commodities are guaranteed with marketability, they are more encouraged to adopt sustainable practices, a way to achieve a pollution-free and healthy environment for farmers. With the rising demand for innovative medicines from natural products, the project’s role of ensuring fair and sustainable circulation of natural and forest commodities leads to equal access to products and by-products of biological commodities of medicinal value. A fair access provides an opportunity to people across countries who are willing to innovate medicines and other pharmaceutical products using the legally traded commodities. This also helps prevent the proliferation of poaching and illegal trade of wildlife as the commodities circulating in global markets are properly monitored and accounted for.

#### SDG 8 Decent Work and Economic Growth

Smallholder farmers and producers in less developed countries are involved in primary production activities from which most of them derive their livelihoods. In these countries, 80% of the farmers are employed directly or indirectly in primary production activities in agriculture, forestry, and natural resources with minimal value addition. The Trade Hub strives on enhancing opportunities for increasing returns to labour, or productive capacity through integration of these farmers into mainstream markets and value addition processes so that they leverage the advantage of collective actions. This brings a fair bargaining power towards better contracts with the government and private sector actors, hence, improving engagement and working conditions in their value chains. As the informal employment in primary production is the mainstay of smallholder producer’s livelihoods, the initiative towards improvement of their working conditions and arrangements will contribute to creating job opportunities and, hence, more prosperous economies.

#### SDG 9 Industry, Innovation, and Infrastructure

The Trade Hub is configured to synergistically work with scientists, researchers, value chain actors, public sectors, and industries across the eight project countries to calibrate and deliver solutions to the challenges across various themes such as markets, trade, regulations, and policies. The intractable challenge is how to sustainably enhance trade and contribute to the well-being of society and humans. Through development of trade and market solutions and models, the review of frameworks and protocols, and the contribution to international trade dialogues, new knowledge is generated and encourages innovations in international trade towards inclusive and sustainable industrialisation. These innovations include tools, frameworks, rules, regulations, policies, and other institutional issues to enhance trade, development, and environment outcomes. Commercialisation of agricultural commodities and wildlife species is promoted as an integrated initiative towards revitalisation of primary industries’ shares of global trade returns. The Trade Hub aims to identify niche markets that allow trade between developing and developed countries including skills and technological transfers.

#### SDG 10 Reduce Equality Within and Among Countries

The Trade Hub helps achieve fair-trade practices both domestically and internationally, with a focus and emphasis on the role of smallholder producers in developing countries in the production and distribution of commodities in global markets. The support and assistance rendered by the project to local partners enable them to produce products in a more sustainable way as well as to protect natural resources and biodiversity, which sustain production. The learning experiences from local partners are shared to the public and policymakers to serve as a guide in formulating an optimal trade policy that provides opportunities to developing countries for trading natural products in a fair and equitable manner. An equal opportunity for trade of commodities leads to inclusive development and growth within and among the participating countries.

#### Goal 12 Responsible Consumption and Production

The Trade Hub promotes sustainable production of agricultural commodities and harvesting of natural resources in a way that does not cause deforestation, species extinction, and illegal trade. Several collaborations with private and public sector institutions in the project countries promote efficient resource use and resilience in the production systems. The project enhances sustainable utilisation of forest resources by encouraging partners across the different countries to strike a balance between conserving swathes of forests and expanding farming areas. This balance is critical for communities to diversify their production activities to guarantee continuous supply of nutritious and healthy food for growing population as well as future generations. Within the value chains covered by the project, private sector companies are encouraged to adopt sustainable practices and achieve a fair-trade footprint allowing marginalised value chain actors and small companies to increase their markup. As an R4D project, it is configured to strengthen scientific and technical capacity of researchers and development practitioners in less developed and emerging economies to move towards more sustainable patterns of consumption and production of agricultural and wildlife products.

#### SDG 13 Climate Action

Working in more than eight commercial value chains and partnering with research institutions and the public sector in the eight countries, the Trade Hub attempts to promote sustainable production by practices that do not contribute to greenhouse gas emissions while building resilience to climate stresses by conserving natural resources and biodiversity. The outputs of the project within the eight countries can be used as models to encourage small-scale production in all parts of the world to adopt climate-smart production techniques and practices. Enhancing the resilience of production systems is achieved through the generation of new knowledge on climate-resilient technologies as most of the agricultural commodities and wildlife species are earmarked for export. The support to primary producers is tailored towards strengthening the adaptive capacity and minimising adverse effects on nature and environments. Some initiatives also ensure that production of agricultural commodities and harvesting of wildlife do not cause degradation of land, species extinction, deforestation, pollution, and other negative impacts on environments. The evidence generated within the hub is utilised to strengthen human and institutional capacities to climate change mitigation and adaptation.

#### SDG 15 Life on Land

The Trade Hub focuses on the dual goal of meeting the demand for agricultural commodities and wildlife species and ensuring sustainable production, utilisation, and harvesting so as to minimise negative economic, social, and environmental impacts. To achieve this, the project promotes value chains for palm oil, rattan, bamboo, rubber, soy, sugarcane, coffee, and wildlife species whose production, harvesting, and trade are aligned with conservation of terrestrial ecosystems. The project ensures stewardship towards environmental conservation and restoration, particularly forests and dryland ecosystems and contributes to the reduction of deforestation, reclamation of degraded forests, and the guarding against poaching, trafficking, and illegal trade of wildlife species and products.

#### Goal 17 Partnerships for Goals

The Trade Hub leverages global partnerships by multi-stakeholder alliances with research institutions, public and private sector organisations, and farmers to deliver its global objective of making trade benefit nature and humans. It forms North–South and South–South collaborations across organisations and researchers in generating evidence and developing solutions to the current global challenges. One of the nine work packages of the project specifically aims to refine trade protocols through participation in global conferences, platforms, and dialogues on non-discriminatory and equitable multilateral trade systems under the World Trade Organization (WTO). Working along the value chains of agricultural and wildlife products, the project contributes to improving export quotas for participating countries with a particular view to enhancing the integration of upstream value chain actors such as smallholders and expanding cooperation across countries and regions.

Mapping of the impacts of the activities undertaken by partners in the Trade Hub shows that the most fulfilled SDGs due to the BWs are the SDG 12 (Sustainable Consumption and Production), SDG 15 (Life on Land), and SDG 2 (Zero Hunger) in descending order.

### Interactions Between SDG Impacts

The 2030 Agenda for Sustainable Development is a call for a transformative step to achieving a sustainable and resilient future with an emphasis on integrated and indivisible outcomes between the different goals. This is one of the reasons why SDG impact evaluation is regarded as difficult, aggravated by the failure of MEL to recognise the systematic nature of SDGs, resulting in silos in project and programmes (IIED 2019). Therefore, there is a need to adopt a holistic and integrated approach through the alignment and cooperation that would bring about the radical, large-scale, and sustainable change implied by the SDG transformation.

**Fig. 5:**
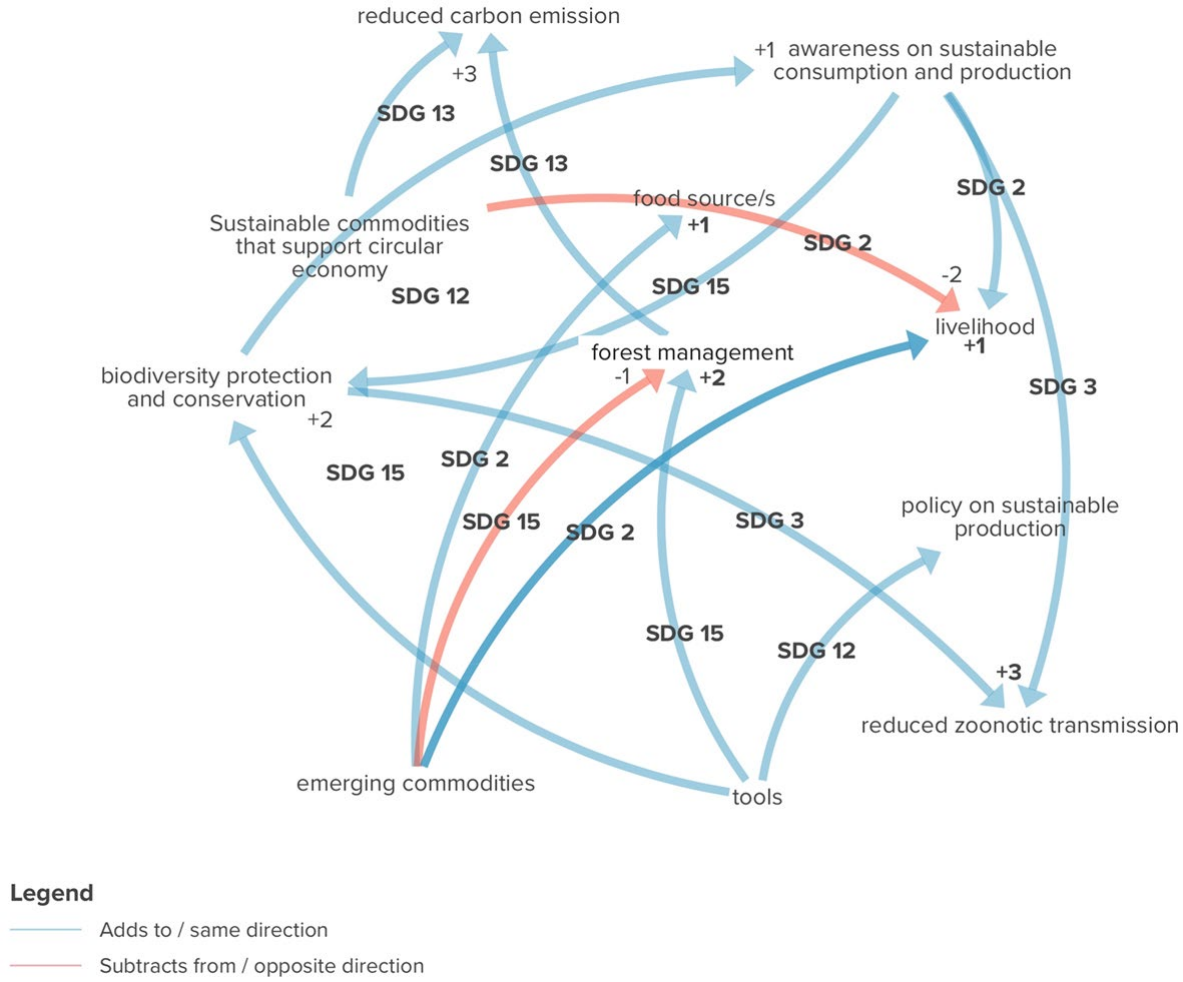
Interaction of the outputs contributing to the SDGs 12, 15, and 2

Using the three core SDGs of the Trade Hub, interaction analysis was performed to determine the type of influence among the outputs of the Trade Hub in relation to the SDGs 12, 15, and 2 (Fig. 5).

The Trade Hub’s role in strengthening the adoption of bamboo and rattan in Indonesia and African states as commodities can support circular economy by promoting sustainable substitute materials across the world. Institutionalising the replacement of high-carbon footprint materials such as plastics would progress towards low-carbon economies and climate change measures (+ 3 to 13.2.1 and 13.2.2). Moreover, the developed supply chain models of the different commodities help emphasise the different implications from local production to global distribution of commodities, thereby making all stakeholders rethink the institutionalisation of sustainable consumption and production. This facilitates the achievement of biodiversity protection and conservation (+ 2 to 15.a.1). However, this shift in commodity priority and production practices may temporarily reduce the yield, potentially affecting the livelihoods of farmers in the short run (− 2 to 1.1.1 and 1.2.1).

The Trade Hub’s work in Indonesia on cocoa using integral metrics/tools enables the identification of the remaining high integrity forest areas and informs areas at risk of conversion into commodity production (+ 1 to 13.1.1 and 13.2.2). Partners working on soybean and palm oil products in Brazil, China, Tanzania, Central Africa, and Indonesia focus on the assessment and monitoring of deforestation in relation to production, which is the basis for enhancing policy for sustainable production (+ 2 to 21.1.1). In addition, the project works with partners in the Congo basin states and Tanzania to enhance the understanding of consumer choices, impacts of wild meat consumption and trade on people and environments, and raise awareness among stakeholders (+ 2 to 12.8.1).

The Trade Hub’s support on value addition in emerging commodities increases the potential for discovering commodities in the wild, which helps address the scarcity of food sources but increase the probability of exploiting these commodities, potentially compromising forest management (− 2 to 15.2.1). The project’s work in the Central African region on emerging commodities with demand assessment and production techniques for bush mango and other crops would help fill the gap in the current farming practices to improve production, which will increase the opportunity to raise yields and income (+ 3 to 1.1.1 and 1.2.1).

The BWs that are major contributors to the three core SDGs of the Trade Hub are BW 1, BW 5, and BW 2 with the weights of 0.86, 0.84, and 0.80, respectively. The weights indicate that the greatest contributions come from farmers’ incentives and empowerment (BW 1), followed by international trade agreements (BW 5) and regulations of nature and social impacts of trade (BW 2). Figure 4 also shows that the SDG 12 (Responsible Consumption and Production) is directly associated to most of the BWs, followed by the SDG 15 (Life on Land) and SDG 2 (Zero Hunger).

### SDG Contribution Analysis

**Fig. 6:**
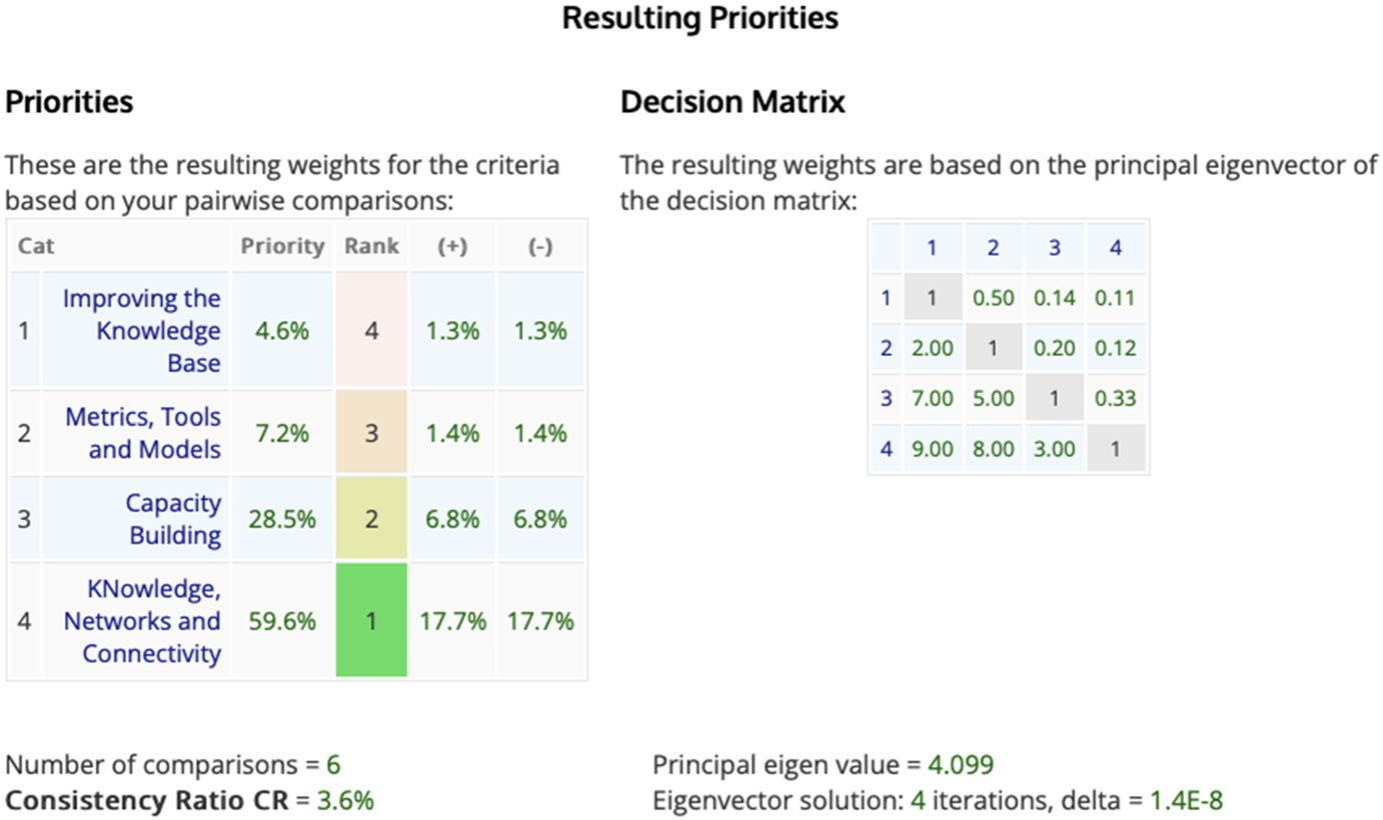
Pairwise comparison between the four ‘mechanisms’ of the Trade Hub

The priority ranking analysis revealed that knowledge, networks, and connectivity was ranked first (56.9%), followed by capacity building (28.5%), metrics, tools, and models (7.2%), and improving the knowledge base (4.6%) (Fig. 6).

**Fig. 7:**
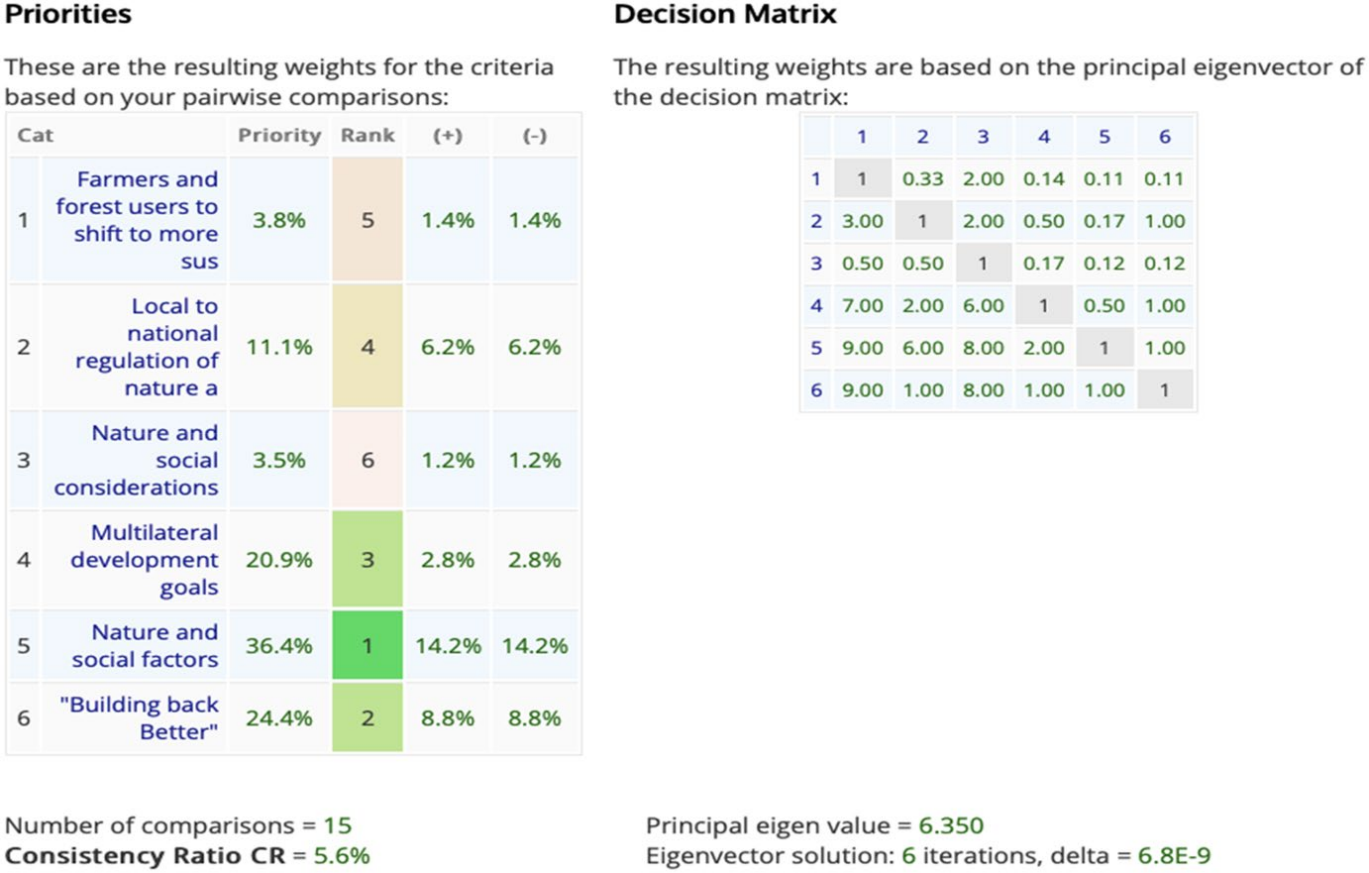
Pairwise comparison between the six Big Wins of the Trade Hub

Similarly, when the respondents were asked to weigh the importance of the BWs, results revealed that BW 5 (nature and social factors are better considered in international trade agreements) as the top priority, followed by BW 6 (building back better post COVID-19 agenda that integrates nature and social considerations of trade) (24.4%) and BW 4 (multilateral development goals linking nature, people and trade are strengthened) (24.4%) (Fig. 7).

The links between environments and trade agreements help regulate natural resource degradation while strengthening the supply chains of commodities to drive economic growth, especially the developing countries. On the other hand, the post COVID-19 agenda gives direction in investment and behavioural changes to reduce the likelihood of stocks and increase resilience.

## Conclusion

This paper aimed to provide a comprehensive review and mapping of SDG evaluation methods applicable for international R4D projects and assessed the applicability of the most frequently used evaluation methods to the UKRI-GCRF Trade Hub project as a case. The AHP was the most popular method because of its simplicity and ease of use. Mapping and evaluation of SDG contributions of the Trade Hub revealed that the six BWs as defined for the project contribute to ten out of the SDGs. The major contributors to these SDGs were BW 1 (Farmers and forest users are empowered to shift to more sustainable practices and obtain fair economic returns), BW 5 (Nature and social factors are better considered in international trade agreements), and BW 2 (Local and national regulation of nature and social impacts of trade is strengthened). Interaction analysis among the outputs within the three core SDGs relevant to the Trade Hub revealed both synergy and tradeoff among them. A greater representation of the interactions can be further made using all the outputs connected to the ten SDGs associated to this project. The approaches to evaluation of SDG contributions adopted by countries, governments, private and non-private sectors, and R4D projects have been evolving. In recent years, various methods emerged in attempts to provide frameworks for establishing a bridge between the project focus areas and the SDGs.

This review has certain limitations. The evaluation method adopted in this study is limited to the AHP in weighing the importance and degree of contributions of the BWs to the different SDGs. No other method was used to validate the result. The analysis and mapping made use of the information reported by the partners in the M&E dashboard of the project, with a reconfirmation through interviews. Moreover, this paper used a simple mapping approach to determining the impact and interactions among the outputs. These milestones are the accomplishments of the project in its 3rd year of implementation. Thus, more outputs can still be collected for evaluation of its SDG impacts. Moreover, due to the limited resources, the interaction analysis focused on the outputs anchored to the three core SDGs of the project. It is suggested that at the end of the project, the confirmed outputs be evaluated for SDG impacts to establish a comprehensive report on the contribution to sustainable development. The robustness of the assessment results should be examined by triangulation through use of at least one other method than the AHP. Moreover, deeper analysis of the relationships and interactions among all the outputs would be desirable to serve as a basis for establishing a unique nexus for wildlife and trade, which can strengthen policy formulation and programme implementation.

In all research articles reviewed for this paper, no gold standard framework was identified for assessing international development projects against the SDGs. Thus, further research is suggested to develop a tool that would capture holistic and synergistic contributions of the target outcomes of projects to sustainable development.

## Data Availability

The data that support the findings of this study are available on request from the corresponding author.
